# Test-Retest Reliability of Postural Control Assessment on Biodex BioSway™

**DOI:** 10.1155/2022/7959830

**Published:** 2022-03-02

**Authors:** Daniel Miner, Brent A. Harper, Stephen Glass, Brooke Martin, Molly Polizotto, S. Montana Hearl, Ellen Turner

**Affiliations:** ^1^Radford University Carilion, Department of Physical Therapy, Roanoke, VA, USA; ^2^Chapman University, Department of Physical Therapy, Irvine, CA, USA

## Abstract

**Background:**

Recent protocols for posturographic assessment of postural control and balance have included head shake test conditions to challenge the vestibular contributions of postural control in an effort to increase the diagnostic accuracy of identifying individuals with impaired balance. However, evidence is limited regarding the test-retest reliability of such assessment protocols.

**Purpose:**

The purpose of this study was twofold: to determine the test-retest reliability of postural control assessment on the Biodex Biosway™, an accessible and field expedient tool for posturographic assessment, and to determine the test-retest reliability of the Head Shake Sensory Interaction and Balance Test (HS-SIB), an adaptation of the modified Clinical Test of Sensory Interaction and Balance (mCTSIB) which adds two head shake conditions to challenge the vestibular contributions to postural control. *Study Design*. This was a correlational time series cohort study completed in a biomechanics laboratory.

**Methods:**

The sample consisted of nineteen healthy adults (10 females, 9 males). Sway Index, Equilibrium Score, and the area of the ellipse enclosing 95% of the anterior-posterior (AP) and medial-lateral (ML) center of gravity (COG) displacement (AREA_95_) are the 3 summary variables. Standard Error of Measurement (SEM) and Minimum Detectable Change (MDC) are also reported.

**Results:**

Test-retest reliability was generally poor with limited exceptions. Moderate to good reliability was observed for the more challenging stance conditions (ICC range 0.58-0.81), including those with head shake.

**Conclusions:**

Field-expedient systems, such as the Biodex BioSway™, may offer reliable posturographic testing where gold-standard methods are not available. Clinicians should be aware that less demanding test conditions have limited reliability; however, test-retest reliability of this assessment tool is improved with more challenged stance conditions and the inclusion of a head shake task.

## 1. Introduction

Presently, computerized dynamic posturography (CDP) represents the gold standard for assessment of balance and postural control. CDP protocols are often administered using the NeuroCom® SMART Balance Master®. This reliable computerized posturography test systematically evaluates sensory interaction for postural control to quantify how deficits and impairments in sensorimotor integration impact postural control; however, such systems are stationary and often cost-prohibitive [1]. CDP protocols may or may not incorporate yaw-plane head rotation conditions [1]. Some reports suggest that inclusion of a head shake test condition may help to increase the diagnostic utility of posturographic assessment of postural control by identifying those with more subtle impairments which may be clinically important but more difficult to detect with standard clinical assessment [2–4].

Recently, portable technologies, such as the Biodex Biosway™, have allowed the clinician to extend some of the functionality of gold-standard testing methods into field-based settings. These systems may lack sensitivity due to deficits in measurement precision or lack of dynamic sway-referencing [5]. The modified Clinical Test of Sensory Interaction and Balance (mCTSIB) systematically alters visual and somatosensory inputs to examine the impact on balance and postural sway [6]. The mCTSIB does not specifically assess how balance is affected by altering vestibular input, which may limit its ability to detect more subtle deficits in balance performance [1, 7]. The Head Shake Sensory Interaction and Balance (HS-SIB), an adaptation of the mCTSIB, combines the low-cost benefit of using the Biodex BioSway™ with the addition of two head shake conditions to challenge the vestibular contributions of postural control [5]. The head shake conditions in the HS-SIB protocol, performed on the Biodex BioSway™, were designed to assess the impact of altering vestibular inputs for postural control without the increased cost and complexity of the head shake sensory organization test (HS-SOT) on the NeuroCom® (see Table 1).

A recent study of the mCTSIB and HS-SIB performed on the Biodex Biosway™ revealed that there is limited validity of this assessment on the Biodex Biosway compared to the gold standard assessment of the sensory organization test or head shake sensory organization test on the NeuroCom Balance Master [5]. However, the head shake conditions in the HS-SIB assessment on the Biodex BioSway™ improved the validity of this assessment compared to the NeuroCom® [5].

Many studies which have utilized the Biodex Biosway™ for assessment of postural sway or compared the Biodex Biosway™ to other devices have reported that this is a reliable assessment tool [8–15]. However, on closer examination, these same studies have supported this claim by citing reliability studies which were actually performed on a separate hardware system designed for dynamic posturography, the Biodex Balance System [16–18]. The reliability of the Biodex Biosway™ has yet to be established. The major difference between the Biodex Biosway and the Biodex Balance System is that the Biodex Biosway™ has a stable platform for static posturography; however, the Biodex Balance System has a dynamic platform for dynamic posturography. The reliability of a dynamic posturography system can not be automatically generalized to a static posturography system as patterns of postural sway will be very different in a static vs. dynamic system. It is also unclear whether or not the raw data from these two systems is sampled at the same rate which may also affect the reliability and validity of this system.

To our knowledge, there are only two papers which have specifically investigated the reliability and validity of the Biodex Biosway™ [5, 13]. Dewan and colleagues [13] found the Biodex Biosway to be a valid tool for postural control assessment across multiple stance conditions including double limb support and single limb support. Miner and colleagues [5] found that the Biodex Biosway had fair reliability in stance conditions on a firm surface as compared to the gold standard for posturographic assessment the NeuroCom Balance Master. However, the assessment of postural sway on stance conditions with a foam pad was not valid compared to the analogous conditions on the Balance Master with a sway-referenced platform.

The purpose of this study was to assess test-retest reliability of the HS-SIB performed on Biodex BioSway™. It is hypothesized that clinically acceptable reliability will be observed in testing conditions involving yaw-plane head rotations. If a correlation is found to exist between test-retest reliability scores for the HS-SIB on the Biodex BioSway™, this will demonstrate the advantage of using the HS-SIB test as a reliable measure and cost-effective option for field-based assessment.

## 2. Methods

### 2.1. Participants

Participants were recruited as a convenience sample via flyers and word of mouth. Individuals between 18 and 60 years old with or without a history of concussion are qualified to be included in the study. Individuals with diagnosed vestibular disorder, neuropathy, or recent (<6 months) leg injury or surgery were excluded from the study. The sample consisted of 19 healthy, active, young adults (10 females, 9 males; age 23-30 (mean 24.8 ± 1.8); height (m) = 1.72 ± 0.11; weight (kg) = 71.74 ± 14.21). All participants signed an informed consent form prior to participation in this study, and data collection was performed under the research protocols established and approved by the Radford University IRB. The authors have no conflict of interest to disclose regarding the equipment being tested in this study. All participants were naïve to the test conditions prior to participation in this study.

### 2.2. Measures

Balance trials were performed on a Biodex Biosway™ force plate. Ground reaction forces were sampled at 20 Hz and used to calculate anterior-posterior (AP) and medial-lateral (ML) center of pressure (COP). COP time histories were stored for offline analysis.

### 2.3. Design and Procedures

On the first day of testing, gender, age, height, and weight were recorded. The history was followed by assessment of the Head Shake Sensory Interaction and Balance (HS-SIB) on the Biodex BioSway™. Participants returned within 7-10 days of initial assessment to repeat the HS-SIB to establish test-retest reliability of this assessment tool.

Participants visited the lab for two separate testing sessions. On both occasions, each participant completed a series of quiet standing balance trials. Participants were barefoot for all tests and maintained a consistent foot position as recorded using a coordinate system printed on the stance surface. A total of 6 trials were completed in each testing session, with each trial lasting 30 sec. The six test conditions were presented in randomized order to minimize any potential impact on test-retest results which may be attributable to test order. (See Table 1 for description of testing conditions.) Participants were instructed to remain as motionless as possible for the duration of the trial, with the exception of yaw-plane head rotations for those trials involving a head shake [5].

For head shake trials, participants rotated their heads continuously in the yaw-plane (i.e., about the vertical axis) 30 degrees to either side at a frequency of 2 Hz. Participants practiced these head shake procedures with the eyes open prior to recording test trials. For familiarization, visual and auditory feedbacks were provided via a head-mounted laser and audible metronome, which provided head shake amplitude and frequency information, respectively. During head shake test trials, for which the participants' eyes were closed, feedback regarding frequency of head rotations was maintained via continued use of the metronome while amplitude feedback was provided verbally by the examiner.

### 2.4. Data Analysis

COP time histories were used to construct angular center of gravity (COG) series, from which we calculated 3 summary variables: (1) the Sway Index (SI), (2) the Equilibrium Score (ES), and (3) the area of the ellipse enclosing 95% of the AP/ML COG displacement (AREA_95_), similar to previous work [5]. The SI (Equation (1)), a two-dimensional root mean square displacement, is an outcome measure described in the Biosway™ reference materials. The ES (Equation (2)) is one of the primary metrics reported from the SOT on the NeuroCom® and effectively describes the proportion of the AP cone of stability one uses during a given trial. Finally, AREA_95_ is a familiar outcome variable in the postural control literature. It is included here as a measure that is relatively robust to the impact of extreme individual data points.

(1) $$\begin{array}{c} \text{SI}=\sqrt{\frac{1}{n}\left(\overset{n}{\underset{i=1}{\sum }}{\left(x_{i}-\overset{-}{x}\right)}^{2}+{\left(y_{i}-\overset{-}{y}\right)}^{2}\right),} \end{array}$$

(2) $$\begin{array}{c} \text{ES}=\frac{12.5^{\circ }-\left(\theta_{\max}^{\circ }-\theta_{\min}^{\circ }\right)}{12.5^{\circ }}*100. \end{array}$$

### 2.5. Statistical Analysis

Test-retest reliability of each outcome was assessed using intraclass correlation coefficients ICC (2, k) for absolute agreement. To provide a more complete picture of the Biodex Biosway™ system's measurement properties, we also report Standard Error of Measurement (SEM) and Minimum Detectable Change (MDC) (see Table 2). All signal processing and statistical computations were performed in R programming language (version 3.6.1; The R Foundation, Vienna, Austria). ICC value reference ranges for reliability interpretation were as follows: <0.5 poor, 0.5-0.75 moderate, 0.75-0.90 good, and >0.90 excellent [19]. Statistical significance was determined as *p* < 0.05.

## 3. Results

Descriptive statistics by test condition (*C*) are presented in Table 2. Reliability, SEM, and MDC are presented in Table 3. Note that HS trials were missing for 1 participant. Reliability for all outcomes was poor in C1, C2, and C3. Poor reliability was also observed for ES in C4. Moderate reliability was observed for all remaining outcomes, with the exception of AREA_95_ in C4, for which reliability was good. For the SI output metric from the Biodex Biosway™, moderate reliability was observed for C4, C5, and C6. Poor reliability was observed for SI in C1, C2, and C3.

## 4. Discussion

The purpose of this study was to evaluate the test-retest reliability of postural control assessment on the Biodex Biosway™ utilizing the HS-SIB. The output metrics of the Biodex Biosway™ provide moderate reliability for the most challenging stance conditions of the HS-SIB where individuals are unable to rely on visual input for balance, and somatosensory input is altered by standing on a foam surface, and/or vestibular inputs are altered through inclusion of head shake. Mathematical models suggest that when accounting for extremes in postural sway, the reliability of this assessment is good for balance conditions where vision and somatosensory inputs are altered or removed. The Biodex Biosway™ demonstrates poor reliability during stance conditions where only one sensory system is being manipulated (e.g., conditions 1-3). This suggests the need for more challenging protocols which manipulate more than one sensory modality at a time and include a head shake component to improve the reliability of this assessment tool (e.g., conditions 4-6).

Clinical utility of this assessment is supported by the Minimum Detectable Change (MDC) scores which indicate the responsiveness of this posturographic assessment to changes in performance. For clinicians, understanding the responsiveness of an assessment tool is critical to inform clinical decision-making with regard to rehabilitation management of individuals with impaired postural control. In an athletic population, current sideline assessments have limited diagnostic accuracy outside of the acute window of time following injury and are not sensitive enough to capture more subtle impairments which may increase risk for future injury with premature return to play [20, 21].

These findings suggest that field-expedient systems, such as the Biodex BioSway™, may offer reliable posturographic testing when gold-standard methods are not available. It should be noted that even though the Biodex BioSway™ may not be sensitive enough to detect lower magnitude postural sway in less challenging stance conditions (e.g., conditions 1-3), the reliability of assessment for condition 2 (EC, firm) and condition 3 (EC, foam) is improved by the inclusion of a head shake task in conditions 5 and 6, respectively.

Previous studies have examined interdevice reliability of postural control assessment, but few have examined test-retest reliability within the Biodex Biosway™ system [5, 13]. Miner et al. [5] assessed the validity of the HS-SIB on the Biodex BioSway™ compared to the gold-standard head shake sensory organization test (HS-SOT) on the NeuroCom® SMART Balance Master® for quantifying postural sway. This study concluded that the two devices should not be used interchangeably given the large observed ranges for 95% levels of agreement (LOA). In some cases, these ranges were attributed to the differences in static posturography (Biodex BioSway™) compared with dynamic posturography (NeuroCom®). The authors also report limitations in the spatial resolution of the Biodex Biosway™, which they conjecture could affect that system's measurement properties in the less challenging conditions [5].

This is the first study conducted to determine test-retest reliability of performing the HS-SIB on the Biodex BioSway™. The poor reliability observed in the less challenging conditions of the HS-SIB (conditions 1-3) is likely related to sway magnitudes approaching the limits of the Biosway™ system's measurement precision. Measurement precision of this system is likely affected by differences in spatial resolution and sampling rates of ground reaction forces—Biodex BioSway™ (20 Hz) versus NeuroCom® (100 Hz). Similar results were also found in a reliability and validity study for the mCTSIB conducted by Dawson et al. [22]. This study did not report ICC data for each individual condition but did report poor test-retest reliability for condition 1 demonstrated by an ICC of 0.24, which aligns with our observations. Inclusion of a head shake component for balance testing on the Biodex Biosway™ may help compensate for lack of precision relative to higher-cost systems and potentially further improve its suitability as a field-expedient measure.

There are several limitations to our study. The sample size was small and included young healthy adults. Therefore, it did not represent a random sample which limits the generalizability of the results. The time of day when testing was performed was not controlled. Despite utilization of a metronome and verbal feedback during familiarization with the head shake protocol, reliability of the postural sway assessment during the head shake conditions may have been affected by a variable degree of challenge to the vestibular system due to minor inconsistencies in the frequency of head rotation between trials.

## 5. Conclusions

The HS-SIB, using the Biodex BioSway™, has a moderate test-retest reliability for conditions with larger postural sway changes. The inclusion of a head shake condition to increase the vestibular challenge may improve the reliability of the HS-SIB assessment and overcome the limitations in measurement precision of the Biodex BioSway™.

## Figures and Tables

**Table 1:** Sensory constraints of each test condition on Biodex BioSway™.

BioSway™	Test condition (C)	Availability of sensory inputs
Modified clinical test of sensory interaction and balance	C1: eyes open, firm surface	Vision, vestibular, and somatosensory all available
	C2: eyes closed, firm surface	Vision removed, vestibular and somatosensory available
	C3: eyes open, foam surface	Vision and vestibular available, somatosensory altered
	C4: eyes closed, foam surface	Vision removed, vestibular available, somatosensory altered

Head shake sensory interaction and balance test	C5: eyes closed, firm surface, headshake 120 degrees per second	Vision removed, vestibular altered, somatosensory available
	C6: eyes closed, foam surface, headshake 120 degrees per second	Vision removed, vestibular and somatosensory altered

**Table 2:** Descriptive summary.

		Descriptive summary		
Condition		Sway Index (cm)	Equilibrium Score	COG A95 (cm^2^)
EO, firm		0.34 (0.14)	95.81 (1.98)	0.40 (0.50)
EC, firm		0.57 (0.19)	92.39 (2.52)	0.79 (0.61)
EO, foam		0.58 (0.11)	92.70 (2.13)	1.24 (0.62)
EC, foam		1.22 (0.26)	83.67 (4.65)	5.13 (2.39)
HS, EC, firm		0.54 (0.19)	93.16 (2.44)	0.75 (0.46)
HS, EC, foam		2.10 (0.60)	70.75 (8.29)	13.26 (8.68)

EO: eyes open; EC: eyes closed; HS: head shake; cm: centimeters; COG A95: area of center of gravity 95% confidence ellipse.

**Table 3:** Reliability analysis and agreement metrics.

	Reliability/agreement metrics						
	ICC (CI)	*F*	Sig.	SEM	MDC	Corr	Sig. (Corr)
EO, firm (C1)							
SI	0.00 (-0.38-0.38)	1.00_(18, 18)_	0.500	0.14	0.39	-0.13	0.59
ES	0.03 (-0.35-0.40)	1.06_(18, 18)_	0.450	1.95	5.42	0.01	0.97
AREA_95_	0.06 (-0.32-0.43)	1.14_(18, 18)_	0.400	0.48	1.34	0.04	0.87
EC, firm (C2)							
SI	0.47 (0.13-0.71)	3.06_(18, 18)_	0.010	0.14	0.38	0.52	0.02
ES	0.46 (0.12-0.71)	2.85_(18, 18)_	0.020	1.85	5.14	0.49	0.03
AREA_95_	0.41 (0.07-0.67)	2.67_(18, 18)_	0.020	0.47	1.31	0.56	0.01
EO, foam (C3)							
SI	0.16 (-0.23-0.50)	1.37_(18, 18)_	0.250	0.10	0.28	0.13	0.59
ES	0.46 (0.11-0.71)	2.73_(18, 18)_	0.020	1.56	4.34	0.48	0.04
AREA_95_	0.00 (-0.38-0.38)	1.00_(18, 18)_	0.500	0.62	1.71	-0.02	0.94
EC, foam (C4)							
SI	0.62 (0.26-0.82)	5.53_(18, 18)_	0.000	0.16	0.45	0.75	0.00
ES	0.45 (0.10-0.70)	3.15_(18, 18)_	0.010	3.46	9.58	0.60	0.01
AREA_95_	0.81 (0.55-0.92)	12.64_(18, 18)_	0.000	1.04	2.88	0.87	0.00
HS, EC, firm (C5)							
SI	0.58 (0.26-0.79)	3.91_(17, 17)_	0.000	0.13	0.35	0.59	0.01
ES	0.62 (0.32-0.81)	4.61_(17, 17)_	0.000	1.54	4.26	0.65	0.00
AREA_95_	0.69 (0.42-0.85)	5.49_(17, 17)_	0.000	0.26	0.73	0.70	0.00
HS, EC, foam (C6)							
SI	0.59 (0.27-0.79)	4.09_(17, 17)_	0.000	0.36	1.00	0.64	0.00
ES	0.63 (0.34-0.82)	4.71_(17, 17)_	0.000	4.53	12.55	0.65	0.00
AREA_95_	0.59 (0.28-0.80)	3.94_(17, 17)_	0.000	5.21	14.44	0.66	0.00

EO: eyes open; EC: eyes closed; C: condition; Firm: stable standing surface; Foam: medium density foam standing surface; HS: head shake; SI: Sway Index; ES: Equilibrium Score; AREA_95_: area of center of gravity 95% confidence ellipse; ICC: intraclass correlation coefficient; CI: confidence interval; Sig: significance; SEM: standard error of the mean; MDC: Minimum Detectable Change.

## Data Availability

The data used to support the findings of this study are available from the corresponding author upon request.

## References

[1] Freeman L., Gera G., Horak F. B., Blackinton M. T., Besch M., King L. (2018). Instrumented test of sensory integration for balance: a validation study. *Journal of Geriatric Physical Therapy (2001)*.

[2] Heick J. D., Bay C., Dompier T. P., Valovich McLeod T. C. (2017). Relationships among common vision and vestibular tests in healthy recreational athletes. *International Journal of Sports Physical Therapy*.

[3] Honaker J. A., Janky K. L., Patterson J. N., Shepard N. T. (2016). Modified head shake sensory organization test: sensitivity and specificity. *Gait & Posture*.

[4] Park M. K., Lim H. W., Cho J. G., Choi C. J., Hwang S. J., Chae S. W. (2012). A head shake sensory organization test to improve the sensitivity of the sensory organization test in the elderly. *Otology & Neurotology*.

[5] Miner D. G., Harper B. A., Glass S. M. (2021). Validity of postural sway assessment on the Biodex BioSway™ compared with the NeuroCom Smart Equitest. *Journal of Sport Rehabilitation*.

[6] Wrisley D. M., Whitney S. L. (2004). The effect of foot position on the modified clinical test of sensory interaction and balance. *Archives of Physical Medicine and Rehabilitation*.

[7] McNerney Kathleen M., Coad M. L., Burkard R. F. (2018). Learning effects and the sensory organization test: influence of a unilateral peripheral vestibular impairment. *American Journal of Audiology*.

[8] Paniccia M., Wilson K. E., Hunt A. (2018). Postural stability in healthy child and youth athletes: the effect of age, sex, and concussion-related factors on performance. *Sports Health*.

[9] Burt L. A., Gabel L., Billington E. O., Hanley D. A., Boyd S. K. (2020). Postural balance effects associated with 400, 4000 or 10,000 IU vitamin D3 daily for three years: a secondary analysis of a randomized clinical trial. *Nutrients*.

[10] Burt L. A., Billington E. O., Rose M. S., Raymond D. A., Hanley D. A., Boyd S. K. (2019). Effect of high-dose vitamin D supplementation on volumetric bone density and bone strength: a randomized clinical trial. *Journal of the American Medical Association*.

[11] Carr W., Yarnell A. M., Ong R. (2015). Ubiquitin carboxy-terminal hydrolase-l1 as a serum neurotrauma biomarker for exposure to occupational low-level blast. *Frontiers in Neurology*.

[12] Corwin D. J., McDonald C. C., Arbogast K. B. (2020). Clinical and device-based metrics of gait and balance in diagnosing youth concussion. *Medicine and Science in Sports and Exercise*.

[13] Dewan B. M., Roger James C., Kumar N. A., Sawyer S. F. (2019). Kinematic validation of postural sway measured by Biodex Biosway (force plate) and SWAY balance (accelerometer) technology. *Bio Med Research International*.

[14] Johansson J., Nordstrom A., Gustafson Y., Westling G., Nordstrom P. (2017). Increased postural sway during quiet stance as a risk factor for prospective falls in community-dwelling elderly individuals. *Age and Ageing*.

[15] Imhoff S., Fait P., Carrier-Toutant F., Boulard G. (2016). Efficiency of an active rehabilitation intervention in a slow-to-recover paediatric population following mild traumatic brain injury: a pilot study. *Journal of Sports Medicine*.

[16] Schmitz R., Arnold B. (1998). Intertester and intratester reliability of a dynamic balance protocol using the Biodex Stability System. *Journal of Sport Rehabilitation*.

[17] Hinman M. R. (2000). Factors affecting reliability of the Biodex Balance System: a summary of four studies. *Journal of Sport Rehabilitation*.

[18] Jaco Ras M., Puckree T. (2014). Injury incidence and balance in rugby players. *Pakistan Journal of Medical Sciences*.

[19] Koo T. K., Li M. Y. (2016). A guideline of selecting and reporting intraclass correlation coefficients for reliability research. *Journal of Chiropractic Medicine*.

[20] Mucha A., Trbovich A. (2019). Considerations for diagnosis and management of concussion. *The Journal of Orthopaedic and Sports Physical Therapy*.

[21] Murray N., Salvatore A., Powell D., Reed-Jones R. (2014). Reliability and validity evidence of multiple balance assessments in athletes with a concussion. *Journal of Athletic Training*.

[22] Dawson N., Dzurino D., Karleskint M., Tucker J. (2018). Examining the reliability, correlation, and validity of commonly used assessment tools to measure balance. *Health Science Reports*.

